# Effects of EPA supplementation on plasma fatty acids composition in hypertriglyceridemic subjects with FABP2 and PPARα genotypes

**DOI:** 10.1186/2251-6581-11-25

**Published:** 2012-12-10

**Authors:** Hamideh Pishva, Mohsen Amini, Mohammad Reza Eshraghian, Saeed Hosseini, Soltan Ali Mahboob

**Affiliations:** 1 Department of cellular, Molecular Nutrition, School of Nutrition Sciences and Dietetics, Tehran University of Medical Sciences, Tehran, Iran; 2 Department of Medicinal Chemistry, Faculty of Pharmacy, and Drug Design & Development Center, Tehran University of Medical Sciences, Tehran, Iran; 3 Department of Epidemiology and Biostatistics, School of Public Health, Tehran University of Medical Sciences, Tehran, Iran; 4 Endocrinology Metabolism Research Center (EMRC), Tehran University of Medical Sciences, Tehran, Iran; 5 Nutrition Research Center, Tabriz University of Medical Sciences, Tabriz, Iran

**Keywords:** Plasma fatty acids composition, Eicosapentaenoic acid, Polymorphism, Fatty acid binding protein-2, Peroxisome proliferator-activated receptor

## Abstract

**Background:**

Fatty acid binding protein 2 (FABP2) and peroxisome proliferator-activated receptor α (PPARα) are involved in cellular uptake and metabolism of fatty acids. Polymorphism of FABP2 and PPARα may influence plasma levels of fatty acids in those who take supplemental eicosapentaenoic acid (EPA). The purpose of this study was to study the potential associations between the Ala54/Thr polymorphism in FABP2 protein and the Leu162/Val in exon 5 and G/C in intron 7 of PPARα with plasma fatty acids composition after EPA supplementation.

**Methods:**

Twenty three FABP2 Ala54 and twenty three Thr54 carriers with hypertriglyceridemia were enrolled in this study. Participants took 2 g of pure EPA daily for 8 wks. Plasma fatty acids composition was determined and changes from the baseline were measured.

**Results:**

Although EPA supplementation increased the level of plasma EPA and ω-3 fatty acids in both carriers of FABP2 and PPARα genes, these effects were more pronounced in Thr54 and Val162 carriers. EPA supplementation decreased the level of some n-6 fatty acids such as arachidonic acid.

**Conclusion:**

EPA consumption has more favorable effects on blood n-3 fatty acids and can change the level of plasma n-3 fatty acids, particularly EPA. Because the FABP2 Thr54 polymorphism appears to be prevalent in hypertriglyceridemic subjects, increasing EPA intake in these subjects could be an effective strategy for preventing cardiovascular diseases. Finally, diets and micronutrient recommendations should be individualized for high risk people.

## Introduction

Dietary fat intake is believed to contribute to development of chronic diseases, in particular cardiovascular disease [1]. No biomarkers reflect the absolute fat intake, however, measuring fatty acids concentrations in various biological samples reflect to some extent, the proportional intake of fatty acids [2]. Fatty acids can be measured as free fatty acids in serum, components of circulating triglycerides, components of erythrocyte membranes, phospholipids or cholesterol esters, or adipose tissue from different sites. The amount of serum or plasma fatty acids reflects the composition of dietary intakes of the past few hours (triglyceride) or the past few days (cholesterol ester and phospholipids fatty acids) [3].

Changes in plasma fatty acids composition reflect abnormalities in lipoprotein metabolism and dietary habits and have been widely studied in many animal and epidemiological [4,5] and clinical human studies [6,7].

Fatty acids of the n-3 family, particularly the long-chain n-3 fatty acids, are important nutrients throughout the life. Several epidemiological studies have shown that n-3 fatty acids in blood differ significantly among individuals [8-10]. This family of fatty acids has been historically associated with a lower risk of cardiovascular disease, including stroke [11] and coronary heart disease [12,13]. In children, cardiovascular benefits have been attributed to long-chain n-3 fatty acids [14,15]. Docosahexaenoic acid (DHA) and eicosapentaenoic acid (EPA) two well-known long-chain n-3 fatty acids are important for their protective effects on cardiovascular disease; increased dietary intake of them has resulted in decreased cardiac mortality in a large secondary prevention study [16].

The composition of serum fatty acids can not only be used as an indicator of dietary fat quality [17], but can also be used as a biomarker for assessing metabolic and cardiovascular disease risk [18,19]. Intestinal fatty acid binding protein 2 (FABP2) is a small cytosolic protein involved in intracellular fatty acid (FA) transfer and metabolism. Peroxisome proliferator-activated receptor α (PPARα) is involved in glucose and lipid metabolism and thus may have a role in development of dyslipidemia, atherosclerosis, obesity, insulin resistance, and type II diabetes mellitus. We have shown that FABP2 genotypes influence the lipid-lowering effects of EPA supplementation in hypertriglyceridemic subjects [20]. We, therefore, conducted this study to determine the potential associations between the Ala54/Thr polymorphism in FABP2 protein and the Leu162/Val in exon 5 and G/C in intron 7 of PPARα with plasma fatty acids composition after EPA supplementation.

## Subjects and methods

### Subjects

Participants were selected from the hypertriglyceridemic subjects referred from Tehran Central Laboratories to Endocrinology and Metabolism Research Center (EMRC). The inclusion criteria were a serum TG level >200 mg/dL (>2.3 mmol/L), and a fasting blood glucose of <110 mg/dL (<6.2 mmol/L). Those who had received lipid lowering agents, oral contraceptive pills, diuretics, sex hormones, thyroid medications, or omega-3 supplement, and patients with a history of gastrointestinal diseases, and smokers were excluded from the study.

After determination of their FABP2 genotypes, the first 23 eligible subjects who were found as Ala54 carriers and the first 23 eligible Thr54 carriers were enrolled in the study. Participants took two grams per day of pure EPA for eight weeks (four gel caps, each containing 500 mg ethyl ester EPA 90%, courtesy of Minami Nutrition, Edegem, Belgium). Two capsules were taken in the morning and two in the evening. The participants were followed weekly at the EMRC; a checklist for weekly consumption of capsules was filled and capsules for the next week were given to them. All of the subjects consumed controlled diet (Percentage of energy from carbohydrate, fat, and protein diets were similar).

A blood sample was drawn from each participant following a 14-hour overnight fasting at the baseline and after eight weeks of EPA supplementation. Height and weight were measured by a Seca scale (Germany) with light clothing and no shoes on. Body mass index (BMI) was then calculated. Waist circumference was measured with a flexible tape midway between the lowest rib and the iliac crest. The hip circumference was measured at the widest part of the gluteal region.

The study was approved by Ethics Committee of EMRC, *Tehran University of Medical Sciences* (TUMS). All participants were informed of the nature of the study and gave a written informed consent. The biochemical analyses were carried out at EMRC laboratory, TUMS. Genetic studies were conducted at the Department of Medical Genetics, TUMS. The plasma fatty acids composition was determined at the Department of Medicinal Chemistry and Pharmaceutical Sciences Laboratory, TUMS.

We calculated the sample size as fallow:

α=0.51−β=0.80P value<0.05

$$n={\left(\left(\left[Z_{1-\alpha /2}\right]+\left[Z_{1-\beta }\right]\right)/d\right)}^{2}\text{d}=0.61$$

n=20

### Laboratory analyses

Plasma samples and sera were separated from blood samples by centrifuging at 4C and 1800 *g* for 15 min and stored in 1-mL aliquots in sterile tubes at -80C until used. Serum and plasma lipid and lipoprotein levels were measured as described previously [20].

### Plasma fatty acid extraction and gas chromatography

Fatty acid extraction was done by Folch method [21] with some modifications. Plasma was homogenized in chloroform: methanol (2:1 vol/vol containing 50 mg/L butylated hydroxy toluene); normal saline was added to the solution, shacked vigorously and allowed for phase separation. The upper layer was drawn off by aspiration and washed several times; the lower phases were collected. Extracted lipids were dried under a stream of nitrogen. The dried lipids were soaponified by the method described previously [22]. Soaponified fatty acids were transesterified by boron trifluoride (BF3) in methanol. BF3 was added to the sample and incubated at 100°C in a water bath for an hour. After cooling to room temperature, hexane, HCl and water were added, shacked vigorously, centrifuged, and the upper phase was taken into a new tube and dried with nitrogen. Before injecting to the instrument, methanol and ethylated margaric acid (as an internal standard) were added to samples. Fatty acids methyl esters (FAMEs) were measured by gas chromatography. A capillary column with 60 m length, 0.25 mm internal diameter and 0.2 μM film thickness on an HP 6890 GC equipped with flame ionization detector was used to qualify and quantify FAMEs. The initial column temperature was set at 195°C for 2 min, which increased to 205°C by increments of 2°C/min, then to 214°C by 1°C/min, then to 240°C by 15°C/min and held for 10 min. Helium was used as the carrier gas at an initial flow rate of 1 mL/min for 8 min, which increased to 1.3 mL/min for 4.2 min and then to 1.9 mL/min. The detector temperature was set at 300°C and the injector temperature at 250°C. FAMEs were identified by comparison with the retention times of Supelco 37 component FAME mix standard. We focused on PUFAs in chromatogram and excluded short- and medium-chain saturated fatty acids from chromatogram. Different concentrations of FAME mix with added ethylated margaric acid were injected to gas chromatography machine (GC) to obtain the standard curve for each fatty acid. The peak area of a given fatty acid was divided by the peak area of the internal standard (ethylated margaric acid) then with respect to the standard curve, concentrations of fatty acids in plasma were estimated.

### Genotyping

#### Ala54Thr (Gene ID: 2169)

Genomic DNA was extracted using the Flexi Gene DNA kit (Qiagen, GmbH, Hilden, Germany) as described previously [20]. A 180-bp DNA fragment containing the G to A nucleotide substitution in exon 2 (codon 54) of the FABP2 gene (Ala54Thr) was genotyped by polymerase chain reaction-restriction fragment length polymorphism (PCR-RFLP) as described previously [20].

#### Leu162Val (gene ID: 55465)

The Leu162Val mutation of the PPARα gene is caused by a C to G transversion at nucleotide 484 in exon 5. The PCR-RFLP method was used as described earlier [23].

#### Intron 7

The PCR-RFLP method was used to determine intron 7 polymorphism (mutation) as described previously [23].

### Statistical analyses

The normality of distribution of continuous variables was tested by one-sample Kolmogorov-Smirnov test. To normalize the continuous variables not normally distributed, a log transformation was applied. The mean plasma fatty acids concentrations between the two study groups with different FABP2 genotypes were compared by independent sample *Student’s t* test.

Since only few subjects with Thr54/Thr were found among the participants, they were pooled with Ala54/Thr subjects and analyses were carried out on the pooled data. Results are presented as Means±SE unless otherwise noted. Analyses were performed by SPSS® for Windows® ver 11.5. A p value <0.05 was considered statistically significant.

## Results

The baseline characteristics of subjects were described previously [20,23]. Table 1 shows the plasma fatty acids compositions in hypertriglyceridemic subjects.

**Table 1:** Plasma fatty acids composition in hypertriglyceridemic subjects

**Fatty Acids**	**Concentration in plasma μg/mL n=46**	**Fatty Acids**	**Concentration in plasma μg/mL n=46**
Miristic acid (C14:0)	20.95±0.6	13,16-Docosadienoic acid (DDA, C22:2 n-6)	8.28±1.2
Palmitic acid (C16:0)	152.07±44.7	alpha-Linilenic acid	4.9±17.9
Stearic acid (C18:0)	105.91±12.1	11,14,17-Eicosatrienoic acid (C20:3 n-3)	4.12±0.9
Arachidic acid (C20:0)	12.4±7.7	Eicosapentaenoic acid (C20:5, n-3)	2.5±1.2
Behenic acid (C22:0)	13.2±1.3	Docosahexaenoic acid (C22:6, n-3)	11.9±8.2
Oleic acid (C18:1)	131.69±20.2	Sum of saturated fatty acids	304.5±32.1
11-Ecosenoic acid(C20-1)	9.41±1.5	Sum of monounsaturated fatty acids	164.79±26.2
Nervonic acid (C24:1)	11.45±5.36	Sum of polyunsaturated fatty acids	360.5±50.2
Linoleic acid (C18:2)	200.5±35.2	Sum of W-6 fatty acids	110.3±218.2
Gamma linolenic acid (C18:3, n-6)	7.4±3.4	Sum of W-3 fatty acids	20.6±11.4
11,14-Ecosadienoic acid (C20:2, n-6)	21.65±6.9	Total fatty acids	777.96±95.6
Dihomo gamma Linolenic acid (C20:3 n-6)	8.27±1.2	Arachidonic/DGLA+EPA	23.7±21
Arachidonic Acid	52.1±23.0	W6:W3 ratio	10.0±1.6

Values are means±SE.

Table 2 shows plasma fatty acids levels of studied subjects stratified by their FABP2 genotypes. Concentrations of EPA (p<0.001), DHA (p<0.055), and some of n-3 fatty acids (p<0.001) were higher in those with Thr54 polymorphism than Ala54 after EPA supplementation. Changes in levels of other fatty acids did not significantly differ between subjects with G or A alleles.

**Table 2:** Plasma fatty acids concentration after 8 weeks of EPA supplementation in hypertriglyceridemic subjects stratified by FABP2 genotypes

**Concentration in plasma μg/mL**									***p***
**Fatty acids**	**Pre-treatment**	**Post-treatment**	***Paired t-test P value***	**Pre-treatment**	**Post-treatment**	***Paired t-test P value***	**Difference between pre- and post-treatment**		
	**Ala54 (n=23)**			**Thr54**^**§**^**(n=23)**			**Ala54 (n=23)**	**Thr54**^**§**^**(n=23)**	
Eicosapentaenoic acid (C20:5, n-3)	1.45±0.4	6.66±0.8	0.001^*^	4.65±1.0	67.96±12.9	0.001^*^	5.20±0.8	61.76±12.3	0.001^**^
Docosahexaenoic acid (C22:6, n-3)	10.47±1.5	16.12±2.0	0.06^*^	22.22±5.9	19.63±1.9	NS	5.65±2.9	29.94±13.1	0.055
Sum of saturated fatty acids	251.03±32.5	286.76±47.4	NS	357.96±43.5	286.76±453.5	0.006^*^	35.72±55.7	153.01±66.8	0.07
Sum of monounsaturated fatty acids	105.38±17.8	173.1±39.6	0.001^*^	224.2±34.5	2306.3±2261.6	0.001^*^	67.72±41.6	2175.3±2157.9	0.07
Sum of polyunsaturated fatty acids	174.93±34.6	322.75±61.5	0.08^*^	442.41±65.8	800.55±74.7	0.05^*^	147.82±81.1	351.38±82.5	0.08
Sum of W6 fatty acids	156.96±32.9	289.0±57.9	NS	403.4±63.6	662.24±65.2	0.001^*^	132.04±76.5	251.55±76.9	NS
Sum of W3 fatty acids	15.97±2.3	33.75±4.1	0.006^*^	38.97±6.9	138.3±16.2	0.05^*^	15.78±5.1	99.82±16.5	0.001^**^
Total fatty acids	531.35±70.5	782.62±138.7	NS	1024.57± 120.7	24326.97± 22645.9	0.005^*^			
W6:W3 ratio	7.65±0.9	7.58±0.9	NS	12.37±2.2	5.54±0.5	0.005^*^	−0.073±1.6	−6.76±2.2	0.02^**^
Miristic acid (C14:0)	20.46±0.6	21.73±0.61	NS	21.46±0.56	23.38±0.96	0.05^*^	1.27±0.96	1.77±0.79	NS
Palmitic acid (C16:0)	104/94±65/2	152.29±30.7	NS	199.2±24.3	319.6±33.5	0.01^*^	47.35±39.3	116.7±40.9	NS
Stearic acid (C18:0)	92.96±12.0	96.13±15.5	NS	118.85±12.0	152.27±25.9	0.05^*^	31.17±18.2	28.5±33.2	NS
Oleic acid (C18:1)	76.95±14.3	143.12±35..2	NS	186.43±27.6	316.83±34.9	0.004	46.17±40.2	124.92±47.3	NS

Values are mean±SEM.

^**§**^Because the number of subjects in the Thr54/Thr group was small, data from the Ala54/Thr group were combined with data from the Thr54 (Ala54/Thr Thr54/Thr) groups.

^**^Significant difference between Ala54 and Thr54 groups.

^*^Significant differences between post- and pre-intervention values.

NS: No significant difference.

Plasma fatty acids levels in hypertriglyceridemic subjects with Val162 polymorphism in PPARα genotypes are shown in Table 3. The concentrations of EPA (p<0.001), and n-3 fatty acids (p<0.001) were significantly higher and the n-6:n-3 ratio (p<0.01) was significantly lower in Val162 than in Leu162 polymorphism. Changes in levels of other fatty acids did not significantly differ between Leu or Val carriers. The levels of EPA (p<0.001) and n-3 fatty acids (p<0.02) were significantly different between GG and GC groups (Table 4).

**Table 3:** Plasma fatty acids concentration after 8 weeks of EPA supplementation in hypertriglyceridemic subjects stratified by PPARα genotypes

**Concentration μg/mL**									**Independent t-test *P value***
**Fatty acids**	**Pre-treatment**	**Post-treatment**	***Paired t-test P value***	**Pre-treatment**	**Post - treatment**	***Paired t-test P value***	**Difference between pre and post treatment**		
	**Leu *(n=38*)**			**Val *(n=8*)**			**Leu *(n=38*)**	**Val *(n=8*)**	
Eicosapentaenoic acid (C20:5, n-3)	3.04±0.7	21.9±3.9	0.001^*^	3.5±1.2	104.35±28.9	0.05^*^	18.9±3.5	100.9±28.5	0.001^**^
Docosahexaenoic acid (C22:6, n-3)	15.5±3.7	28.7±7	0.01^*^	19.03±4.3	58.7±16.3	NS	13.15±7.8	39.63±17.4	
Sum of saturated fatty acids	288.5±26	367.0±37.8	NS	371.53±60.6	542.9±113.5	NS	78.5±44.6	171.38±139.4	0.05^**^
Sum of monounsaturated fatty acids	152.36±22.5	277.1±38.5	0.01^*^	219.5±51.7	596.6±59.3	NS	542.9±41.7	594.4±59.3	NS
Sum of polyunsaturated fatty acids	259.3±37.5	482.2±58.2	0.01^*^	509.23±60.1	887.18±149.4	0.05^*^	222.94±66.6	378.9±127.7	NS
Sum of W6 fatty acids	231.98±35.2	416.5±51.2	0.01^*^	476.5±129.7	707.5±135.6	NS^*^	184.6±61.6	231.1±120.3	NS
Sum of W3 fatty acids	27.32±4.3	65.73±9.5	0.001^*^	32.76±8.6	180.65±26.8	0.01	38.3±9.1	147.89±25.5	0.001^**^
Total fatty acids	700.16±79.1	1126.34±128	0.01^*^	1100.28±202.8	61063.12±594.5	NS	426.18±142.9	5999.85±594.7	NS
Arachidonic/DGLA+EPA	2.68±1.0	1.41±0.2	NS	112.8±109.8	0.89±0.2	NS	−1.3±0.9	−11.9±109.8	NS
W6:W3 ratio	8.59±0.8	6.89±0.6	0.05^*^	15.91±5.5	4.57±0.9	NS	−1.7±1.1	−3.5±1.5	0.01^**^

Values are means±SE.

^**^Significant difference between Leu and Val groups.

^*^Significant differences between post- and pre-intervention values.

NS: No significant differences.

**Table 4:** Plasma fatty acids concentration after 8 weeks of EPA supplementation in hypertriglyceridemic subjects stratified by PPARα (GG/GC) genotypes

**Concentration μg/mL**									**Independent t-test*p value***
**Fatty acids**	**Pre-treatment**	**Post-treatment**	**Pairedt-test*p value***	**Pre-treatment**	**Post-treatment**	**Paired t-test *p value***	**Difference between pre- and post-treatment**	**Difference between after and before values**	
	**GG *(n=24*)**			**GC *(n=22)***			**GG *(n=24)***	**GC *(n=22*)**	
Eicosapentaenoic acid (C20:5, n-3)	1.83±0.62	15.35±5.2	0.001^*^	4.49±0.9	60.24±12.4	0.001^*^	12.52±4.9	55.74±12.1	0.001^**^
Docosahexaenoic acid (C22:6, n-3)	11.26±1.7	26.49±6.6	0.04^*^	21.31±5.9	42.38±11.6	0.003^*^	15.23±6.9	21.07±12.8	NS
Sum of saturated fatty acids	262.08±35.3	243.09±53.9	NS	247.81±30.9	459.06±51.9	<0.009^*^	81.01±52.2	111.25±60.4	NS
Sum of monounsaturated fatty acids	112.44±20.1	217.36±42.9	0.03^*^	218.71±33.7	229.6±22.6	0.002^*^	103.9±42.0	227.4±22.6	NS
Sum of polyunsaturated fatty acids	237.53±57.6	437.02±81.5	NS	277.32±56.6	684.23±79.2	0.001^*^	199.53±99.8	206.9±93.3	NS
Sum of W6 fatty acids	217.9±55.7	382.97±73.0	NS	329.88±53.6	562.5±67.0	0.001^*^	165.08±92.0	222.62±83.0	NS
Sum of W3 fatty acids	19.62±2.9	54.1±11.1	0.009^*^	37.46±6.8	121.73±17.4	0.001^*^	34.45±11.2	84.27±18.7	0.02^**^
Total fatty acids	613.03±92.3	997.5±172.1	0.06^*^	942.4±116.2	24105.8±226.5	<0.001^*^	384.47±17.04	23161.9±226.4	NS
Arachidonic/DGLA+EPA	42.24±39.9	1.54±0.2	NS	2.14±0.5	1.08±0.2	NS	−41.7±39.9	−1.06±0.6	NS
W6:W3 ratio	9.65±2.2	7.56±0.9	NS	102.26±1.1	5.3±0.5	0.007^*^	−2.09±2.4	−4.97±1.3	NS

Values are means±SE.

^**^Significant difference between GG and GC carriers.

^*^Significant difference between post- and pre-intervention values.

NS: No significant difference.

## Discussion

We found that EPA supplementation could increase the level of plasma EPA, in both FABP2 and PPARα genotypes with more effects on subjects with either Thr or Val162 alleles. These results are in keeping with the hypothesis which indicates that presence of the Thr54 allele may increase the binding affinity of FABP2 to long-chain fatty acids (LCFAs) [24]. Furthermore, enhanced intestinal absorption of fatty acids, higher levels of plasma lipids and the consequent enhanced lipid oxidation rates would inhibit *in vivo* tissue sensitivity to insulin. It was later confirmed, in a healthy white population with normal glucose tolerance, that the Thr54 allele was associated with insulin resistance [25].

In fact, in subjects with Thr54 allele, EPA supplementation results in absorption of EPA by entrocytes, which leads to a higher plasma EPA concentration. Although EPA is the precursor of DHA, we did not observe any increase in plasma level of DHA which might be due to poor enzymatic conversion of EPA to DHA. Arterburn, *et al*., previously reported that n-3 fatty acids consumption increased their levels in plasma. They showed that supplementation of adults with 4 g/day pure EPA ethyl ester results in significant increase in EPA concentration in whole plasma and plasma or serum phospholipids, but no increase was seen in DHA concentration, which is consistent with retro conversion of DHA to EPA [26-28]. In the present study, the levels of some fatty acids in plasma were changed. After EPA supplementation, the level of EPA, n-3 fatty acids, MUFA, PUFA and some saturated fatty acids such as miristic, palmitic, oleic, and stearic increased in both The54 and Ala54 carriers, the increase was more pronounced in Thr54 groups. These results were approved the Thr54 hypothesis which states increased fatty acids uptake and transport by Thr54 carriers. King, *et al.*, reported that with consuming two different fat diets the level of fatty acids composition would be different [29]. There are some evidence that the level of n-6 fatty acids will decrease after consumption of n-3 fatty acids [26,30-32]. In the current study, the level of some plasma n-6 fatty acids such as arachidonic acid (AA) decreased in both Ala54 and Thr54 carriers after EPA supplementation; although we could not observe any interaction between EPA consumption and genotype. A decrease in the level of plasma AA after n-3 consumption has been reported previously [32-34]. Hlavaty, *et al.*, reported that supplementing diet with n-3 fatty acids decreases plasma level of some n-6 fatty acids [35]. Berstad, *et al.*, reported that n-3 supplementation decreases plasma AA level [33]. Polymorphism in codon 54 had no significant effect on serum fatty acids composition in adults Finns [36]. In Pima, no significant difference between the long-chain fatty acids amount in adipose and muscle tissues was observed between Ala54 and Thr54 carriers [37]. One study showed that in obese children who were Thr54 carriers, EPA consumption decreased the amount of plasma AA level. In the current study, AA concentrations were lower in Thr54 than Ala54 carriers after EPA supplementation. A decrease in n-6:n-3 fatty acids ratio was observed in both FABP2 and PPARα genotypes after EPA supplementation too. Although the n-6:n-3 fatty acids ratio decreased in both FABP2 and PPARα genotypes, these effects were more pronounced in Thr54 and Val162 than in Ala54 and Leu162 carriers. On the other hand, the ratio of AA: EPA decreased in both Thr54 and Ala54 after EPA supplementation, but no significant differences were observed between the two carriers. For nucleus receptors n-3 fatty acids are stronger ligands than n-6 fatty acids. In Greenland and Japanese people this ratio decreased in both Thr54 and Ala54 carriers after EPA supplementation [38,39].

There is increasing scientific evidence that genetic factors, conferring either protection or risk, also contribute importantly to the incidence of these diseases. SNPs are of particular interest because they can influence disease in a complex but largely unknown manner by interacting with environmental and lifestyle factors.

We showed that EPA supplementation could change the blood fatty acids composition, and thus it could be beneficial for lowering some plasma fatty acids. Since we observed more pronounced changes in blood fatty acids in Thr and Val than in Ala and Leu carriers, we suggest EPA supplementation to be used based on people genotypes.

In conclusion, EPA consumption has more favorable effects on blood n-3 fatty acids and can change the level of plasma n-3 fatty acids, particularly EPA. Because the FABP2 Thr54 polymorphism appears to be prevalent in hypertriglyceridemic subjects, increasing EPA intake in these subjects could be an effective strategy for preventing cardiovascular diseases. Finally, for high-risk people diet and micronutrients recommendation should be individualized.

## Competing interests

All authors declare that they have no conflict of interests.

## Authors’ contributions

HP contributed to conception of the idea and study design, interpretation of data, performing all experiments and writing the manuscript. MA provided assistance in study design of the GC analysis. MRE helped with statistical analysis and interpretation of data. SH helped in editing the manuscript. SAM provided assistant in the design of the study. All authors have read and approved the final form of the manuscript.
